# Characterization of cell-free breast cancer patient-derived scaffolds using liquid chromatography-mass spectrometry/mass spectrometry data and RNA sequencing data

**DOI:** 10.1016/j.dib.2020.105860

**Published:** 2020-06-16

**Authors:** Göran Landberg, Emma Jonasson, Anna Gustafsson, Paul Fitzpatrick, Pauline Isakson, Joakim Karlsson, Erik Larsson, Andreas Svanström, Svanheidur Rafnsdottir, Emma Persson, Daniel Andersson, Jennifer Rosendahl, Sarunas Petronis, Parmida Ranji, Pernilla Gregersson, Ylva Magnusson, Joakim Håkansson, Anders Ståhlberg

**Affiliations:** a Department of Laboratory medicine, Institute of Biomedicine, Sahlgrenska Academy, Sahlgrenska Cancer Center, University of Gothenburg, SE-41390 Gothenburg, Sweden; b Department of Medical Biochemistry and Cell biology, Institute of Biomedicine, Sahlgrenska Academy, University of Gothenburg, SE-41390 Gothenburg, Sweden; c RISE, Research Institutes of Sweden, Bioscience and Materials – Medical Device Technology, SE- 50115 Borås, Sweden; d Wallenberg Centre for Molecular and Translational Medicine, University of Gothenburg, SE-41390 Gothenburg, Sweden; e Department of Clinical Genetics and Genomics, Sahlgrenska University Hospital, SE-41390 Gothenburg, Sweden

**Keywords:** Breast cancer, Patient-derived scaffolds, Extracellular matrix, Liquid chromatography-mass spectrometry/mass spectrometry, RNA sequencing, 3d cell culture

## Abstract

Patient-derived scaffolds (PDSs) generated from primary breast cancer tumors can be used to model the tumor microenvironment *in vitro*. Patient-derived scaffolds are generated by repeated detergent washing, removing all cells. Here, we analyzed the protein composition of 15 decellularized PDSs using liquid chromatography-mass spectrometry/mass spectrometry. One hundred forty-three proteins were detected and their relative abundance was calculated using a reference sample generated from all PDSs. We performed heatmap analysis of all the detected proteins to display their expression patterns across different PDSs together with pathway enrichment analysis to reveal which processes that were connected to PDS protein composition. This protein dataset together with clinical information is useful to investigators studying the microenvironment of breast cancers. Further, after repopulating PDSs with either MCF7 or MDA-MB-231 cells, we quantified their gene expression profiles using RNA sequencing. These data were also compared to cells cultured in conventional 2D conditions, as well as to cells cultured as xenografts in immune-deficient mice. We investigated the overlap of genes regulated between these different culture conditions and performed pathway enrichment analysis of genes regulated by both PDS and xenograft cultures compared to 2D in both cell lines to describe common processes associated with both culture conditions. Apart from our described analyses of these systems, these data are useful when comparing different experimental model systems. Downstream data analyses and interpretations can be found in the research article “Patient-derived scaffolds uncover breast cancer promoting properties of the microenvironment” [1].

Specifications table

**Table:** 

Subject	Cancer research
Specific subject area	Tumor microenvironment in breast cancer
Type of data	Deposited raw data Table Figures
How data were acquired	Mass spectrometry data were acquired using tandem-mass-tag (TMT)-labeled relative quantification LC-MS/MS using the reversed-phase nanoLC interfaced QExactive followed by Orbitrap Tribrid Fusion MS instrument and relative quantification using Proteome Discover database. RNA sequencing data were acquired using an Illumina NextSeq500 instrument. Sequence alignments were performed using STAR with the hg19 reference genome and read counts were performed with HTSeq.
Data format	Raw Analyzed Filtered
Parameters for data collection	Mass spectrometry: 15 breast cancer tumors were collected after surgery from Department of Pathology at Sahlgrenska University Hospital, Sweden. Tumors were decellularized, resulting in cell-free PDSs. RNA sequencing: MCF7 and MDA-MB-231 cells cultured in 2D, PDS, and mouse xenograft model systems. Three to 6 samples of each culture system were analyzed.
Description of data collection	Mass spectrometry: PDSs were homogenized and lysed. Protein analysis was performed with 2 injections using LC-MS/MS. Relative protein levels for each sample were compared to a reference generated from all PDSs. RNA sequencing: libraries were prepared from extracted RNA with the Smart-seq2 protocol. Illumina sequencing was performed followed by alignment using STAR with the hg19 reference genome and read counts with HTSeq.
Data source location	Proteomics were performed at Proteomics Core Facility, Gothenburg University, Sweden. RNA sequencing were performed at TATAA Biocenter. Gothenburg, Sweden.
Data accessibility	Mass spectrometry proteomics data: Repository name: ProteomeXchange Consortium (http://proteomecentral.proteomexchange.org) via the PRIDE partner repository [2] Data identification number: PXD018367 RNA sequencing gene expression data: Repository name: NCBI's Gene Expression Omnibus [3] Data identification number: GSE148483 Direct URL to data: https://www.ncbi.nlm.nih.gov/geo/query/acc.cgi?acc=GSE148483
Related research article	G. Landberg, P. Fitzpatrick, P. Isakson, E. Jonasson, J. Karlsson, E. Larsson, A. Svanström, S. Rafnsdottir, E. Persson, A. Gustafsson, D. Andersson, J. Rosendahl, S. Petronis, P. Ranji, P. Gregersson, Y. Magnusson, J. Håkansson, A. Ståhlberg, Patient-derived scaffolds uncover breast cancer promoting properties of the microenvironment, Biomaterials 235 (2020) 119,705. https://doi.org/10.1016/j.dib.2020.105455

## Value of the data

• The mass spectrometry dataset provides information about differentially expressed proteins in patient-derived cell-free breast cancer scaffolds with associated clinical data.
• The RNA sequencing data provides information about the induced gene expression changes in breast cancer cell lines MCF7 and MDA-MB-231 in response to experimental model systems, including 2D, PDS and mouse xenografts.
• These datasets can benefit researchers interested in breast cancer research, tumor microenvironment and 3D model systems.
• Provided datasets can be analyzed together with most other mass spectrometry and RNA sequencing data.

## 1 Data description

The uploaded mass spectrometry dataset contains raw data files and MSF file outputs with the protein contents of 15 PDSs [1]. Table 1 shows sample information, including tumor grade, estrogen and progesterone status, cell proliferation (Ki67), histological subtype, TMT-labels and sample set details.

**Table 1:** Clinical information and sample details about patient-derived scaffolds (PDSs) used for mass spectrometry analysis.

PDS	Tumor grade (II-III)	Tumor grade (Low-High)	Estrogen receptor	Progesterone receptor	Ki67 staining (%)	Histological subtype	TMT 10-plex label	Sample set
PDS1	III	High	Positive	Negative	70–80	Ductal	127 C	1
PDS2	II	Low	Positive	Positive	20	Ductal	127 N	1
PDS3	III	High	Negative	Negative	40	Ductal	127 N	2
PDS4	III	High	Positive	Positive	57	Ductal	128 C	1
PDS5	III	High	Positive	Negative	90	Ductal	128 C	2
PDS6	II	Low	Positive	Positive	30	Ductal	128 N	2
PDS7	III	High	Positive	Positive	40	Ductal	128 N	1
PDS8	III	High	Negative	Negative	30–40	Ductal	129 C	1
PDS9	III	High	Positive	Positive	40	Ductal	129 C	2
PDS10	II	Low	Positive	Positive	35	Lobular	129 N	2
PDS11	II	Low	Positive	Positive	25	Lobular	130 C	2
PDS12	III	High	Negative	Negative	89–90	Ductal	130 C	1
PDS13	II	Low	Positive	Positive	20	Lobular	130 N	2
PDS14	III	High	Negative	Negative	90	Ductal	130 N	1
PDS15	III	High	Positive	Negative	80	Other	131	1

**Fig. 1:**
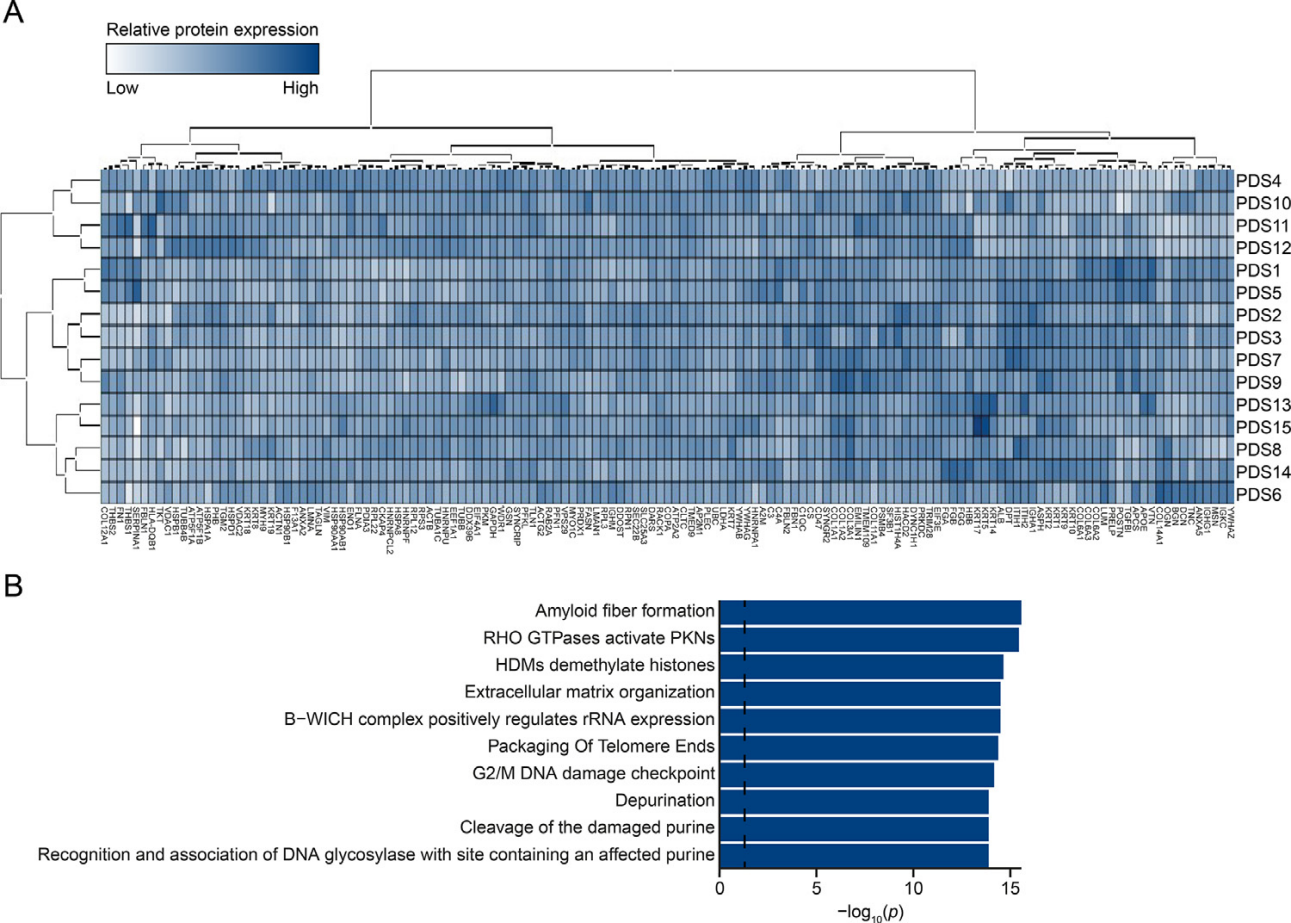
Protein expression in 15 patient-derived scaffolds (PDSs). (A) Heatmap analysis and hierarchical clustering of protein expression, including 143 proteins. (B) Bar-graph representing the top 10 enriched Reactome pathways associated with the 143 detected proteins based on *q*-value. Dashed line indicates *q* = 0.05.

Fig. 1A illustrates the relative protein expression for the 15 different PDSs using heatmap analysis. Hierarchical clustering analyses grouped both PDSs and proteins based on protein expression distribution. Fig. 1B shows the analysis of the identified proteins using Reactome pathway enrichment analysis highlighting various processes, including “Amyloid fiber formation”, “HDMs demethylate histones” and “Extracellular matrix organization” among the ten most significantly enriched pathways.

The uploaded RNA sequencing dataset include raw data in the form of bam files for each samples and a complete read count matrix for all transcripts and samples. Sequencing was performed on extracted RNA samples from MDA-MB-231 and MCF7 cells, each cultured in conventional 2D conditions (*n* = 6), in PDSs (*n* = 3, all PDS samples are from different breast tumors), or as xenografts in mice (*n* = 3).

**Fig. 2:**
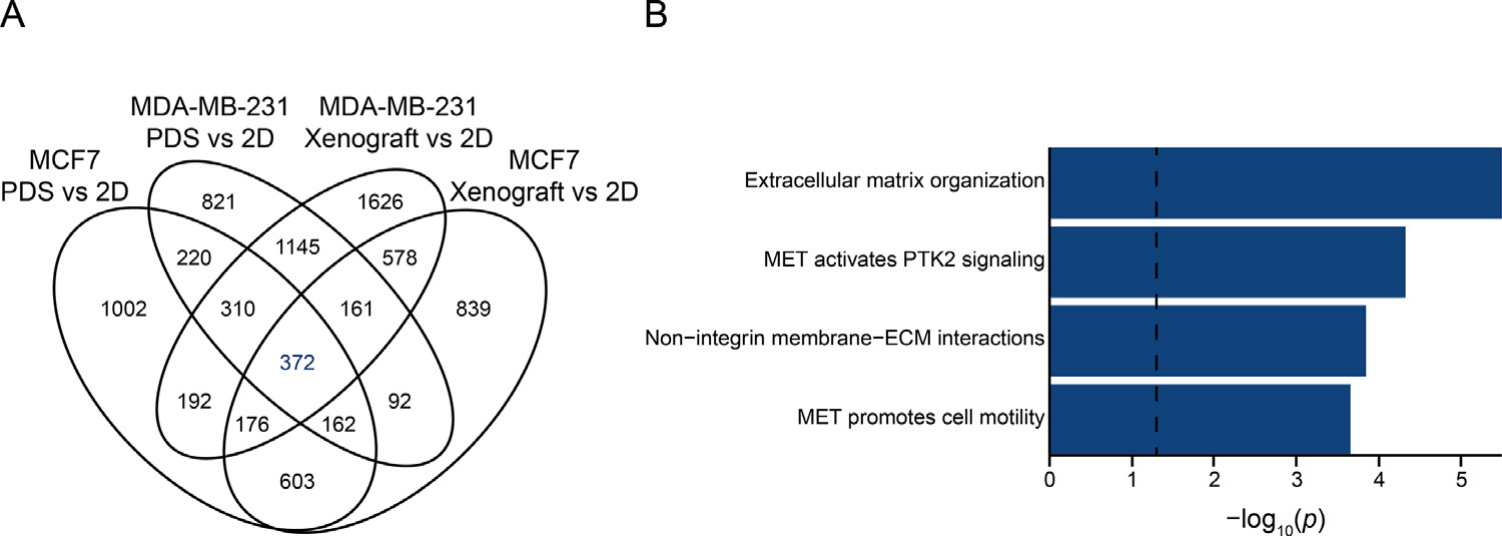
Overlap of significantly regulated genes between culture systems and breast cancer cell lines. (A) Venn diagram illustrating the number of significantly regulated genes between cells cultured in patient-derived scaffolds (PDS) or xenograft compared to 2D using breast cancer cell lines MCF7 and MDA-MB-231, as well as overlap between the systems. (B) Enriched Reactome pathways associated with the 372 genes commonly regulated between PDS and 2D, as well as between xenograft and 2D, in both cell lines. Dashed line indicates *q* = 0.05.

Fig. 2A shows the overlap of regulated genes in the different culture systems and the two breast cancer cell lines. Pathway enrichment analysis of the 372 genes that were commonly regulated in response to PDS and xenograft culture for both breast cancer cell lines demonstrated enriched terms related to both extracellular matrix and cell motility (Fig. 2B).

## 2 Experimental design, materials, and methods

### 2.1 Collection and decellularization of tumors

Fresh primary breast cancer tumors were retrieved directly after surgery via the clinical pathology diagnostic unit at Sahlgrenska University Hospital. Processing of these patient materials and data has been approved by the Regional Research Ethics Committee in Gothenburg (DNR: 515-12 and T972-18). Clinical information about the tumors used for mass spectrometry can be found in Table 1.

Each tumor tissue was sectioned into approximately 3 × 3 × 2 mm pieces. These pieces were decellularized by two 6 h incubations in a lysis buffer containing 0.1% SDS (Sigma-Aldrich), 0.02% sodium azide (VWR), 5 mM 2H_2_O—Na_2_-EDTA (Sigma-Aldrich) and 0.4 mM phenylmethylsulfonyl fluoride (Sigma-Aldrich) followed by a 15 min wash step in the same buffer without SDS. This was followed by a 72 h wash in dH_2_O which was exchanged every 12 h to remove cell debris and a 24 h wash in PBS (Medicago). After washing, patient-derived scaffolds (PDSs) were sterilized for 1 h in room temperature in 0.1% peracetic acid (Sigma-Aldrich) followed by a 24 h wash at 37 °C in PBS containing 1% Antibiotic-Antimycotic (Thermo Fisher Scientific). Wash steps were performed in a 10 L Incushaker (Benchmark) at 37 °C and 175 rpm. Patient-derived scaffolds were kept at 4 °C in a storage buffer containing PBS with 0.02% sodium azide and 5 mM 2H_2_O—Na_2_-EDTA.

### 2.2 Mass spectrometry

Patient-derived scaffolds were prepared by homogenization and lysis in urea, 4% 3-[(3-cholamidopropyl) dimethylammonio]−1-propanesulfonate, 0.2% SDS, 5 mM EDTA (Thermo Fisher Scientific). Liquid Chromatography–Mass Spectrometry/Mass Spectrometry was performed by the Gothenburg University Proteomics Core Facility using 30 µg protein of each PDS. After protein trypsination, peptides were labelled with tandem-mass-tags (TMTs), where each sample and reference received a unique tag. Samples where then separated into two sets, which each was injected twice to the machine (See Table 1). Subsequently, peptides were separated by strong cation exchange chromatography and where thereafter fractioned for mass-to-charge ratio of the peptides. Reverse-phase nanoLC was conducted using QExactive (Thermo Fischer Scientific). Stepped high-energy collision dissociation induced fragmentation was performed using Orbitrap Tribrid Fusion quadruple MS instrument, for peptide sequence information and relative quantification. Further, the Proteome Discoverer database was used for protein identification and relative quantification for the MS-raw data for each merged dataset. Reporter ion intensity ratios in the MS3 spectra were used for quantification of peptides. A reference pool was generated from excess material of all PDSs, from which the relative expression was calculated. Finally, only peptides which were exclusive for that specific protein were considered for quantification. The mass spectrometry proteomics data have been deposited to the ProteomeXchange Consortium via the PRIDE [2] partner repository with the dataset identifier PXD018367. Heatmap analysis and hierarchical clustering was performed in GenEx (MultID). Pathway enrichment analysis was performed using the “enrichPathway” function of the ReactomePA [4] R package v1.30.0 with a significance cutoff of *q* < 0.05 (Benjamini-Hochberg correction).

### 2.3 Cell culture

MDA-MB-231 (ATCC) cells were cultured in complete RPMI-1640 media containing 10% fetal bovine serum, 1% penicillin/streptomycin, 1% sodium pyruvate and 1% l-glutamate (all Thermo Fisher Scientific). MCF7 (ATCC) cells were cultured in complete Dulbecco's modified Eagle's medium (DMEM) media containing 10% fetal bovine serum, 1% penicillin/streptomycin, 1% essential amino acids and 1% l-glutamate (all Thermo Fisher Scientific). Cells were maintained in 37 °C and 5% CO_2_.

### 2.4 Recellularization of patient-derived scaffolds

Breast cancer cell lines, MDA-MB-231 or MCF7, were cultured in PDSs for subsequent RNA sequencing analysis. Before recellularization, PDSs were soaked in complete cell culture media for 1 h at 37 °C to remove residual storage buffer. Cells (3 × 10^5^) were added to 48-well culture plates (Thermo Fisher Scientific) containing PDS pieces in complete media supplemented with 1% Antibiotic-Antimycotic (Thermo Fisher Scientific). After 24 h, PDSs were transferred to new wells and during continued culture, PDSs were transferred again to new wells with fresh media if cells were growing outside the PDS, determined by visual inspection every fourth day. PDSs were cultured for 21 days before RNA extraction.

### 2.5 Xenografts

For xenograft culture, MDA-MB-231 and MCF7 cells were dissociated with Accutase (Sigma Aldrich) and resuspended in DMEM (Lonza) mixed 1:1 with growth factor-reduced Matrigel (BD Biosciences). Cells were injected subcutaneously in the flanks of NOG mice (immunocompromised, non-obese, severe combined immune deficient interleukin-2 chain receptor γ knockout mice, Taconic). For MCF7 cells, a 17β-Estradiol 90 day release pellet (Innovative Research of America) was implanted in the mice 2 to 4 days before injection of cells. Tumors were grown for 32 days before RNA extraction. Mice were housed in Experimental Biomedicine Animal Unit, University of Gothenburg and the study was approved by the Animal Research Ethics Committee of Gothenburg, and proper animal experimentation guidelines were followed (DNR: 5.8.18–10,029/2019 and 141–2014).

### 2.6 RNA extraction

Control cells cultured in 2D conditions were washed with PBS and either frozen on dry ice and stored in −80 °C or directly harvested by scarping off the cells from the culture surface. Cells were lysed in a lysis buffer containing 1 µg/µl bovine serum albumin and 2.5% glycerol (Thermo Fisher Scientific) or in QIAzol (Qiagen). Lysed samples were frozen on dry ice and stored in −80 °C or forwarded immediately to RNA extraction. Cells grown in PDSs were harvested after being washed twice in PBS followed by lysis in 1 µg/µl bovine serum albumin and 2.5% glycerol supplied with RNA Spike II (TATAA) and 4 U/µL RNaseOUT (Thermo Fisher Scientific). To retrieve RNA from tissue samples, samples were thawed on ice prior to homogenization. For PDS and xenograft samples, homogenization was performed using stainless steel beads in TissueLyzer II (both Qiagen) for 2 × 5 min using 25 Hz. Additional 5 min homogenization steps were added until homogenization was achieved, determined by visual inspection. Samples were centrifuged at 4 °C, 10,000 rpm for 1 min and then initially purified by phenol-chloroform extraction followed by extraction using miRNeasy Mini Kit, including DNase treatment (both Qiagen). RNA concentration was measured by NanoDrop (Thermo Fisher Scientific) and RNA quality was randomly assessed with Agilent RNA 6000 Nano Kit using 2100 Bioanalyzer (both Agilent), according to the manufacturer's instructions.

### 2.7 RNA-sequencing library preparation

Extracted RNA samples from 2D, PDS or xenograft cultures were diluted in 1 µg/µl bovine serum albumin, 2.5% glycerol (Thermo Fisher Scientific) and 0.2% Triton X-100 (Sigma-Aldrich) to 10 ng per 5 µl. RNA sequencing libraries were prepared according to the Smart-seq2 protocol [5] with minor changes.

Briefly, for reverse transcription, 1 µM adapter-ligated oligo-dT (5′-AAGCAGTGGTATCAACGCAGAGTACT30VN-3′, Sigma-Aldrich), 1 µM dNTP (Sigma-Aldrich) and ERCC spike-in controls (corresponding to 1 µl of 1:5000 diluted stock solution; Thermo Fisher Scientific) was added to each 5 µl sample followed by a hybridization step at 72°C for 3 min. Concentrations indicated refer to the final reverse transcription reaction. Subsequently, 1x SuperScript II first-strand buffer (Thermo Fisher Scientific), 1 M betaine (Sigma-Aldrich), 5 mM DTT (Thermo Fisher Scientific), 10 mM MgCl2 (Thermo Fisher Scientific), 0.6 µM template switching oligo (TSO;5′-AAGCAGTGGTATCAACGCAGAGTACATrGrG+*G*-3′ with rG = riboguanosine and +*G* = locked nucleic acid modified guanosine; Eurogentec), 15 U RNaseOUT (Thermo Fisher Scientific), and 150 U SuperScript II enzyme (Thermo Fisher Scientific) was added to each sample to a total volume of 15 µl followed by reverse transcription at 42 °C for 90 min and 70 °C for 15 min before being chilled to 4 °C. Complementary DNA samples were stored at −20 °C.

For preamplification, 1x KAPA Hifi HotStart Ready Mix (KAPA Biosystems) and 60 nM adapter PCR primer (5′-AAGCAGTGGTATCAACGCAGAGT-3′, Sigma-Aldrich) was added to 7.5 µl cDNA to a total volume of 50 µl followed by preamplification at 98 °C for 3 min followed by 24 cycles of amplification at 98 °C for 20 s, 67 °C for 15 s, and 72 °C for 6 min, and a final incubation at 72 °C for 5 min before being chilled to 4 °C. Quality assessment as well as determination of size distribution and concentration was performed using the High Sensitivity DNA Kit on a 2100 Bioanalyzer (both Agilent).

For library preparation, the Nextera XT DNA Sample Preparation and Index kits (both Illumina) were used, according to the manufacturer's recommendations with minor changes. To each sample containing 0.1 ng preamplified cDNA, 10 µl TD buffer and 5 µl ATM was added to a total volume of 20 µl followed by tagmentation at 55 °C for 5 min. Thereafter, 5 µl NT buffer was added and tagmentation was stopped by incubation at room temperature for 5 min (all solutions supplied in the Nextera XT DNA Sample Preparation Kit). For indexing and PCR amplification, 15 µl NMP PCR master mix solution (Nextera XT DNA Sample Preparation Kit) and 5 µl each of i5 and i7 index primers (Nextera XT v2 Index Kit) were added to a total volume of 50 µl followed by PCR amplification at 72 °C for 3 min, 95 °C for 30 s followed by 16 cycles of amplification at 95 °C for 10 s, 55 °C for 30 s, and 72 °C for 30 s, and a final incubation at 72 °C for 5 min before being chilled to 10 °C.

Samples were purified using Agencourt AMPure XP beads (Beckman Coulter), according to the manufacturer's instructions with minor changes. Beads were added to samples to a sample:beads volume ratio of 0.6 followed by incubation at room temperature for 5 min and incubation on a magnetic stand (DynaMag 96 Side, Life Technologies) for another 5 min. After removal of supernatant, beads were washed twice with 200 µl 80% ethanol (Thermo Fisher Scientific) before being air dried. DNase/RNase-free water (Thermo Fisher Scientific) was added to retrieve purified cDNA followed by 2 min incubation in room temperature and on magnetic stand to yield 15 µl eluate. Samples were stored at −20 °C. Mean fragment length was assessed using the High Sensitivity DNA Kit on a 2100 Bioanalyzer and concentration was assessed using the dsDNA High Sensitivity Assay Kit on a Qubit instrument (both Thermo Fisher Scientific) and libraries were pooled equimolarly. Quality and concentration of the final pool was assessed as above and it was diluted to 10 nM before being forwarded to sequencing.

### 2.8 RNA sequencing and data analysis

Sequencing was performed at TATAA Biocenter on a NextSeq 500 instrument using 2 × 150 bp paired-end sequencing. Alignment of sequencing reads were performed with STAR [6], including the SortedByCoordinate option, using the hg19 reference genome and GENCODE V17 reference annotation [7]. ERCC spike-in sequences were included. Read count was performed with HTSeq [8], including the “-s no” and “-m intersection-strict” options. Aligned data in the form of bam files as well as a read count matrix of all samples have been deposited in NCBI's Gene Expression Omnibus (GEO) database [3] and are accessible through GEO series accession number GSE148483.

Differential expression analysis was performed with DESeq2 R package [9] including a pre-filtering to remove genes with a read sum of zero or one. Differentially expressed genes between culture conditions within each cell lines were defined based on log2 (fold change) above 1 or below −1 and an adjusted p-value below 0.05. A Venn diagram was created using the VennDiagram R package v1.6.20. Pathway enrichment analysis was performed as previously described for the protein analyses, using the ReactomePA R package with a significance cutoff of *q* < 0.05.

## Declaration of Competing Interest

GL and AS are board members and shareholders of Iscaff Pharma, and AS is a shareholder of TATAA Biocenter. The patient-derived scaffold approach and data are patent pending. Remaining authors declare that they have no other known competing financial interests or personal relationships which have, or could be perceived to have, influenced the work reported in this article.

## Appendix Supplementary materials

Image, application 1
